# TbSAXO is a MAP6-related protein involved in motility of *Trypanosoma brucei* flagellum

**DOI:** 10.1186/2046-2530-1-S1-P16

**Published:** 2012-11-16

**Authors:** M Bonhivers, D Dacheux, N Landrein, M Thonnus, G Gilbert, A Sahin, H Wodrich, DR Robinson

**Affiliations:** 1 CNRS, Microbiologie Fondamentale et Pathogenicite, UMR-5234, France; 2 University of Bordeaux, Microbiologie Fondamentale et Pathogenicite, UMR-5234, France; 3 Institut Polytechnique de Bordeaux, Microbiologie Fondamentale et Pathogenicite, UMR-5234, France

## 

The microtubules (MTs) of most vertebrate tissue cells will disassemble at low temperature, but some remain cold-stable or resistant to drugs such as nocodazole. It has been shown that MT cold- and nocodazole-resistance is largely due to the association with the class of Microtubule Associated Proteins (MAP) known as MAP6 (previously named STOP for Stable Tubule Only Polypeptide) [1]. MAP6 proteins are expressed only in vertebrates, and have been localized in neurons, astrocytes, oligodendrocytes, fibroblasts, and several tissues. In eukaryotes, the MT-based organelles centrioles, cilia and flagella MT have cold-resistant MTs, but, so far, MAP6 proteins have not been characterized in these organelles. We have recently identified TbSAXO (for Stop AXOneme), a novel flagellar protein in the protozoan parasite *Trypanosoma brucei*. We show here that TbSAXO is a microtubule stabilizing protein with properties similar, upon cold and nocodazole treatment, to those of the microtubule-stabilizing Mn domains of the MAP6 proteins, thus identifying the first MAP6-related protein in a protozoan. Further, we demonstrate, in the parasite, that TbSAXO is an axoneme-associated protein, which plays a role in flagellum motility. We also show that TbSAXO is the first member of a group of MAP6-related proteins (that we named SAXO proteins) present only in organisms with centrioles / cilia / flagella and ranging from protozoa to mammals, suggesting potential roles of the SAXO proteins in cilia and flagella function.

http://mcmp.aquitaine.cnrs.fr/mfp/team_bct_en.php
